# Valproate reduces neuroinflammation and neuronal death in a rat chronic constriction injury model

**DOI:** 10.1038/s41598-018-34915-5

**Published:** 2018-11-07

**Authors:** Jun-Yih Chen, Li-Wen Chu, Kuang-I Cheng, Su-Ling Hsieh, Yung-Shun Juan, Bin-Nan Wu

**Affiliations:** 1 Division of Neurosurgery, Fooyin University Hospital, Pingtung, Taiwan; 2 School of Nursing, Fooyin University, Kaohsiung, Taiwan; 3 Department of Pharmacology, Graduate Institute of Medicine, College of Medicine, Kaohsiung Medical University, Kaohsiung, Taiwan; 4 Yuh-Ing Junior College of Health Care and Management, Kaohsiung, Taiwan; 5 Department of Anesthesiology, School of Medicine, College of Medicine, Kaohsiung Medical University, Kaohsiung, Taiwan; 6 Department of Anesthesiology, Kaohsiung Medical University Hospital, Kaohsiung, Taiwan; 7 Department of Pharmacy, Kaohsiung Medical University Hospital, Kaohsiung, Taiwan; 8 Department of Urology, College of Medicine, Kaohsiung Medical University, Kaohsiung, Taiwan; 9 Department of Urology, Kaohsiung Municipal Ta-Tung Hospital, Kaohsiung, Taiwan; 10 Department of Medical Research, Kaohsiung Medical University Hospital, Kaohsiung, Taiwan

## Abstract

Valproate (VPA) is a well-known drug for treating epilepsy and mania, but its action in neuropathic pain is unclear. We used a chronic constriction injury (CCI) model to explore whether VPA prevents neuropathic pain-mediated inflammation and neuronal death. Rats were treated with or without VPA. CCI + VPA rats were intraperitoneally injected with VPA (300 mg/kg/day) from postoperative day (POD) 1 to 14. We measured paw withdrawal latency (PWL) and paw withdrawal threshold (PWT) 1 day before surgery and 1, 3, 7, 14 days after CCI and harvested the sciatic nerves (SN), spinal cord (SC) and dorsal root ganglia (DRG) on POD 3, 7, and 14. PWL and PWT were reduced in CCI rats, but increased in CCI + VPA rats on POD 7 and POD 14. VPA lowered CCI-induced inflammatory proteins (pNFκB, iNOS and COX-2), pro-apoptotic proteins (pAKT/AKT and pGSK-3β/GSK-3β), proinflammatory cytokines (TNF-α and IL-1β) and nuclear pNFκB activation in the SN, DRG and SC in CCI rats. COX-2 and pGSK-3 proteins were decreased by VPA on immunofluorescence analysis. VPA attenuated CCI-induced thermal and mechanical pain behaviors in rats in correlation with anti-neuroinflammation action involving reduction of pNFκB/iNOS/COX-2 activation and inhibition of pAKT/pGSK-3β-mediated neuronal death from injury to peripheral nerves.

## Introduction

Valproate (VPA), a common and popular anticonvulsant currently widely used for epileptic seizures and bipolar disorders, was initially synthesized in 1882^1^. Its pharmacological effects involve diverse mechanisms that affect the transmission of nerve signals *in vitro* and *in vivo*. VPA is neuroprotective in some neurological diseases^2,3^. VPA was also found to decrease the excitation of neurons by N-methyl-D-aspartic acid (NMDA), inhibit voltage-gated sodium (Nav) channels, and modify the firing of neuronal cells^4^. Some recent studies have found that VPA reduces histone deacetylase (HDAC) activity and stimulates neuronal differentiation of neural stem cells^5^.

Several neuropathic pain models mimic clinical pain conditions of various etiologies. It is generally agreed that rat sciatic nerve chronic constriction injury (CCI) is the most popular animal model of peripheral neuropathic pain^6^. This model is commonly used to produce a cutaneous sensory threshold following sciatic nerve injury in rats and to demonstrate hyperalgesia to harmful heat stimuli and allodynia to noxious mechanical stimuli^7^.

Protein kinase B (also known as AKT) and glycogen synthase kinase-3 (GSK-3) family kinases participate in neuronal apoptosis related to neuronal development and neurodegeneration. The PI3K/AKT pathway is implicated in the modulation of cell functions, including proliferation, differentiation, apoptosis and glucose metabolism^8^. GSK-3β is profoundly expressed in the central nervous system (CNS), and its expression is also associated with several pathologic conditions, including Alzheimer’s disease, schizophrenia and bipolar disorders^9^. GSK-3β is reportedly a critical regulator of toll-like receptor (TLR) signaling to modulate the balance between proinflammation and anti-inflammation in the central and peripheral nervous system (PNS)^10^. Participating in copious pathways through the CNS, GSK-3β affects either neuronal protection or degeneration based on its phosphorylation sites^11^. GSK-3 is also a chief regulator of neuronal progenitor homeostasis during embryonic development^12^.

Activation of the neuroimmune system leads to the induction and persistence of neuropathic pain following nerve injury^13^. Nuclear factor-kappa B (NFκB) is a key modulator of inflammatory processes in glial and neuronal cells^14^. In a recent study transgenic inhibition of glial NFκB reduced inflammation and pain behavior after nerve injury in rats^15^. The underlying mechanism could be attributed to the suppression of NFκB activation and subsequent inhibition of neuroimmune activation associated with the prolongation of neuropathic pain^13^. Therefore, the functional role of NFκB in the neuropathic pain mechanism seems critically important.

Other reports demonstrated that chronic treatment with VPA restricted the conversion of arachidonic acid (AA) into inflammatory factors via cyclooxygenase (COX) in rat brain^16^, and VPA also lowered the DNA binding activity of NFκB and the expression of COX-2 mRNA in the rat frontal cortex^17^. The findings indicated that VPA acts by targeting the arachidonic acid cascade, possibly functionally hyperactive in mania^18^. In the context of neurosurgery, the effect of VPA resembles COX-2 inhibitors in downregulating COX-2 in the parenchyma of rat brain. Whether VPA has any analgesic effect in the peripheral nerve injury model deserves further investigation.

## Results

### Pain behavioral responses after CCI with or without VPA treatment

**Figure 1:**
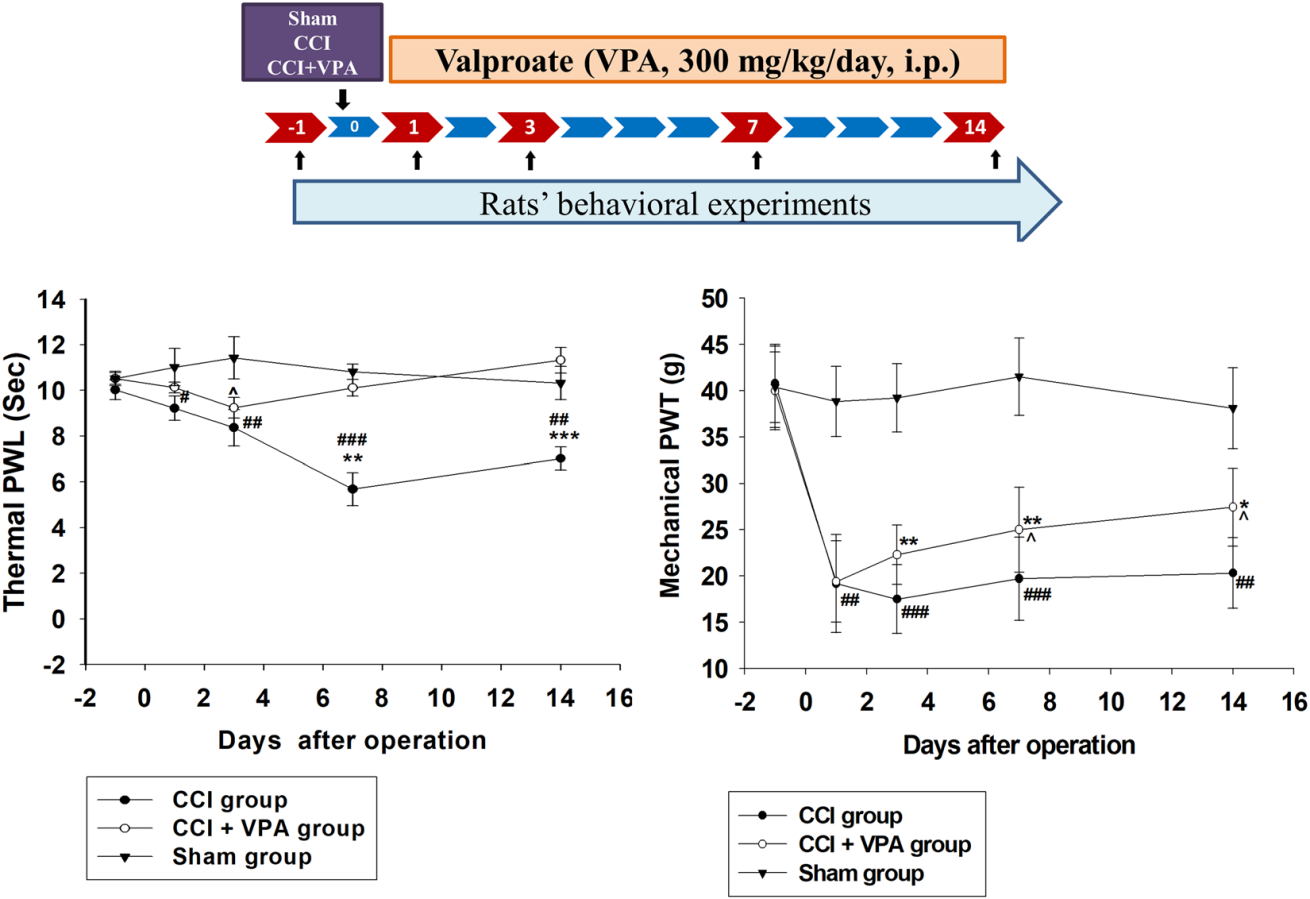
Effects of valproate (VPA) on paw withdrawal latency (PWL) and paw withdrawal threshold (PWT) evoked by chronic constriction injury (CCI) in rats. The top panel depicts the experimental design for the CCI model in rats. Rats were anesthetized with pentobarbital (40 mg/kg) for CCI surgery (day 0) to determine the effect of CCI + VPA (300 mg/kg/day, i.p.) on thermal PWL and mechanical PWT evoked by chronic constriction injury (CCI). PWL and PWT were estimated by the thermal stimulation and paw pressure test, respectively, applied before (day -1) and on days 1, 3, 7 and 14 after surgery. Sham-operated (Sham) rats were subjected to the same surgical procedure, without manipulation of the nerve. Data represent the mean ± SEM for 6 rats per group. ^#^p < 0.05, ^##^p < 0.01, and ^###^p < 0.001 compared to the sham group; ^*^p < 0.05 and ^**^p < 0.01 compared to the CCI group.

The baseline nociceptive latency or threshold was comparable in all groups (p > 0.05, n = 6 in each group, Fig. 1). The CCI group showed markedly less PWL and mechanical PWT from postoperative day (POD) 1 to POD 14, implying that CCI rapidly induced nociceptive pain (<POD 3) and ongoing neuropathic hyperalgesia and allodynia pain behaviors. CCI neuropathic pain was particularly evident on POD 3, becoming most severe pain on day 7 based on thermal PWL and mechanical PWT. PWL or PWT levels showed no significant changes during the experimental protocol in the sham group. The CCI with VPA (300 mg/kg/day, i.p.) treatment group had sharply increased PWL (POD 7 to POD 14) and PWT (POD 3 to POD 14) compared to the CCI group (Fig. 1). Both thermal hyperalgesia and mechanical allodynia induced by CCI improved greatly after repeated VPA administration. VPA appears to attenuate neuropathic pain caused by peripheral nerve injury.

### pNFκB, iNOS and COX-2 expression of in the SN, DRG and SC with VPA treatment

**Figure 2:**
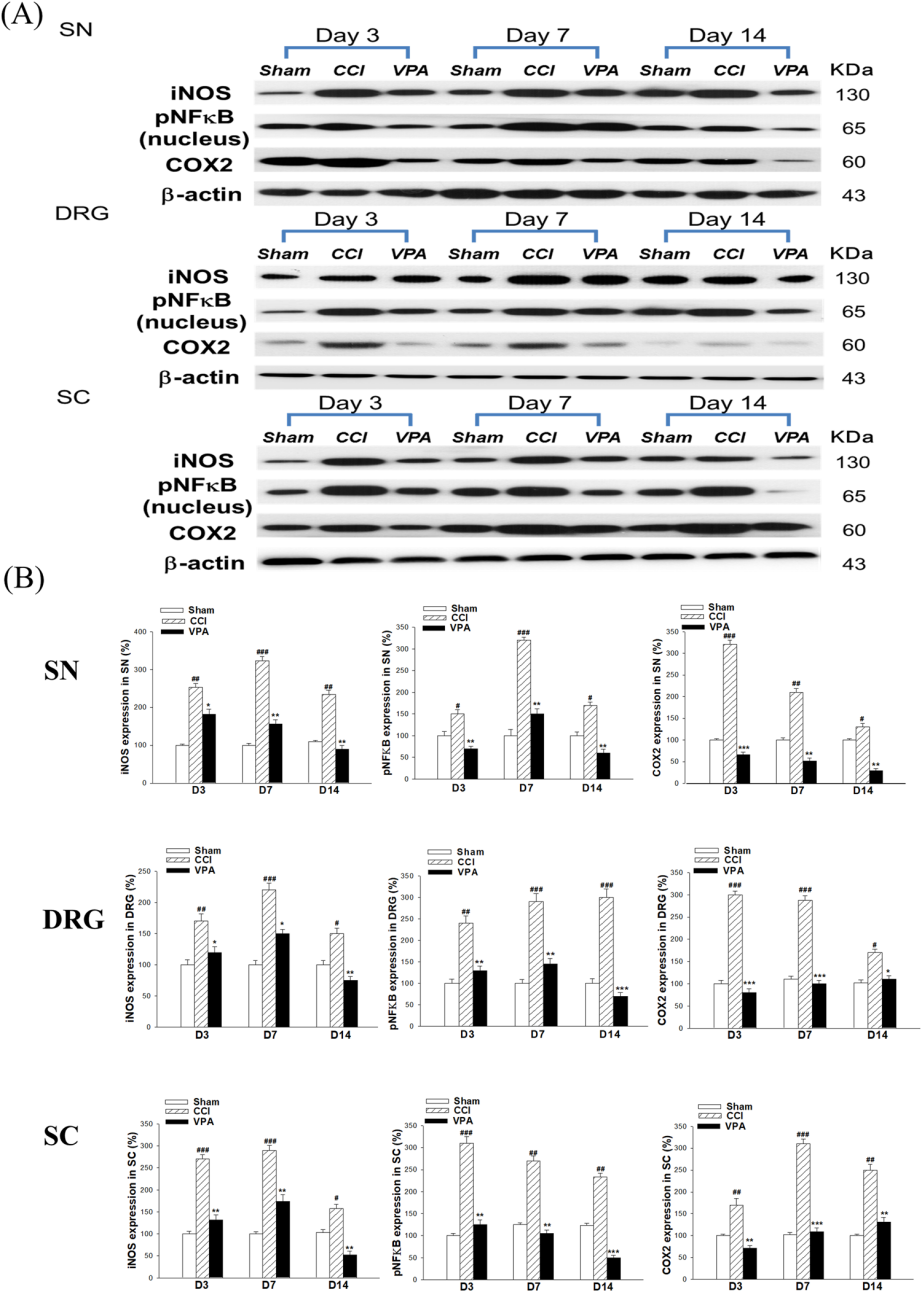
Effects of valproate (VPA, 300 mg/kg/day, i.p.) on the expression of iNOS, pNFκB and COX-2 proteins in the sciatic nerve (SN), dorsal root ganglia (DRG) and spinal cord (SC) induced by chronic constriction injury (CCI). (**A**) Western blots for iNOS, pNFκB and COX-2 proteins from the SN, DRG and SC at days 3, 7 and 14 after CCI. pNFκB expression was measured from nuclear extracts. β-actin was used as the internal control. (**B**) The band intensity was quantified by densitometry and indicated as the relative percentage change compared to the sham group. Data represent the mean ± SEM for 6 rats per group. ^#^p < 0.05, ^##^p < 0.01 and ^###^*p* < 0.001 compared with the sham group; ^*^p < 0.05, ^**^p < 0.01 and ^***^p < 0.001 compared with the CCI group at the corresponding time points.

The rapid increase of CCI group pNFκB and iNOS levels started on POD 3, peaked on POD 7 and remained significantly higher on POD 14. However, after VPA treatment, the protein levels of nuclear pNFκB and iNOS were markedly decreased in the SN, DRG and SC on POD 3, 7 and 14. COX-2 is a well-known pNFκB target gene. COX-2 expression was roughly similar, peaking on POD 3 and gradually reducing on POD 7 and 14 in the SN, DRG and SC after CCI surgery. The CCI + VPA group showed significant reduction of COX-2 expression on POD 3, 7 and 14 in all 3 tissues compared to the sham group (Fig. 2).

### Expression of pAkt/Akt and pGSK-3β/GSK-3β in the SN, DRG and SC related to VPA treatment

**Figure 3:**
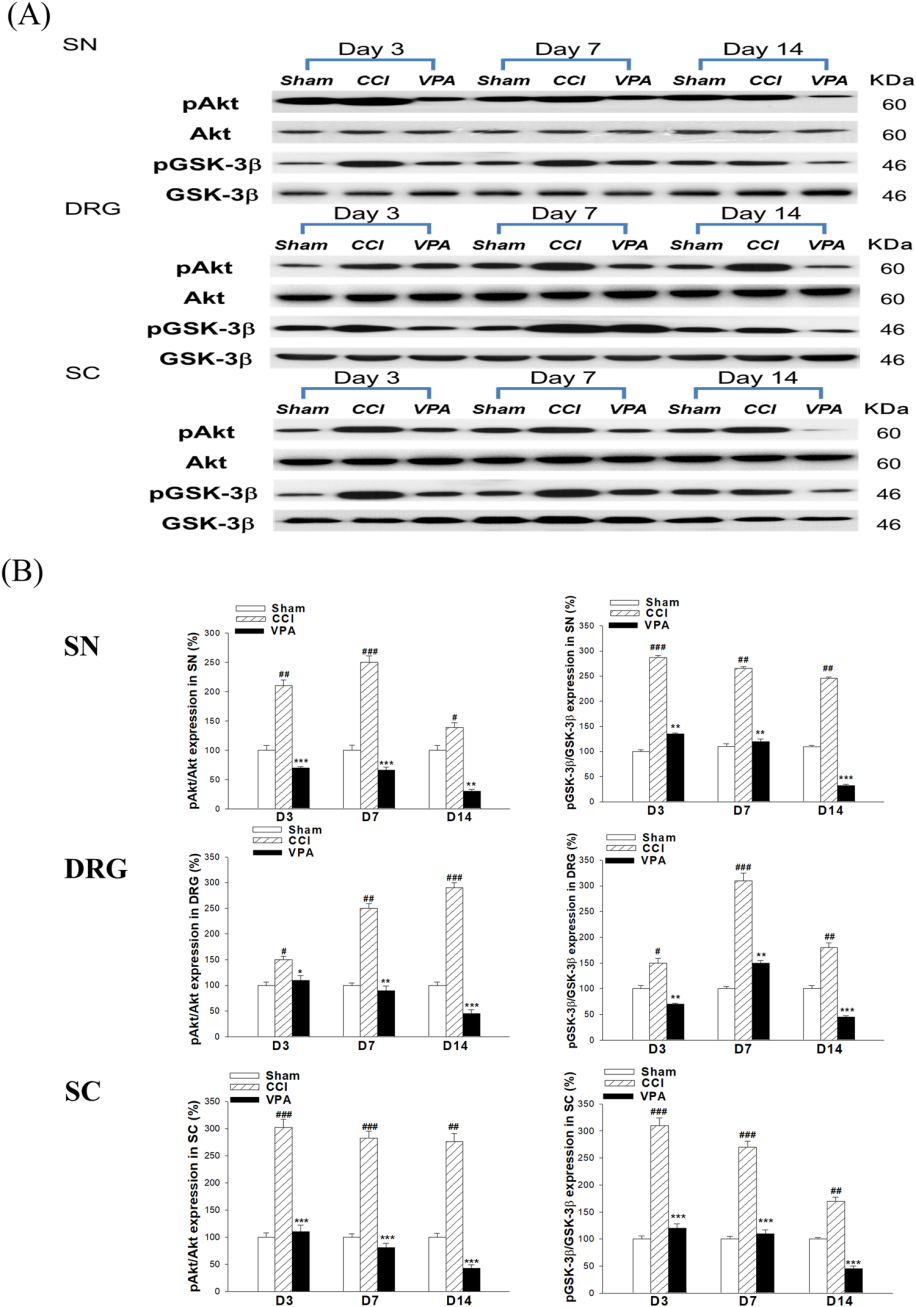
Effects of valproate (VPA, 300 mg/kg/day, i.p.) on the expression of pAKT/AKT and pGSK-3β/GSK-3β proteins in the sciatic nerve (SN), dorsal root ganglia (DRG) and spinal cord (SC) induced by chronic constriction injury (CCI). (**A**) Western blots for pAKT/AKT and pGSK-3β/GSK-3β proteins from the SN, DRG and SC. (**B**) The band intensity was quantified by densitometry and indicated as the relative percentage change compared to the sham group. Data represent the mean ± SEM for 6 rats per group. ^#^p < 0.05, ^##^p < 0.01 and ^###^*p* < 0.001 compared with the sham group; ^*^p < 0.05, ^**^p < 0.01 and ^***^p < 0.001 compared with the CCI group at the corresponding time points.

We observed that CCI elicited a rapid (POD 3) and sustained (POD 14) increase in pAkt and pGSK-3β proteins in the SN, DRG and SC. Both proteins reached the highest point at POD 7 after CCI in each group. Nevertheless, Akt and GSK-3β at each time point disclosed no significant difference between SN, DRG and SC. VPA exerted significant effects on pAkt and pGSK-3β protein in all three tissues for each time point (Fig. 3).

### VPA decreased TNF-α and IL-1β in the SN, DRG and SC

**Figure 4:**
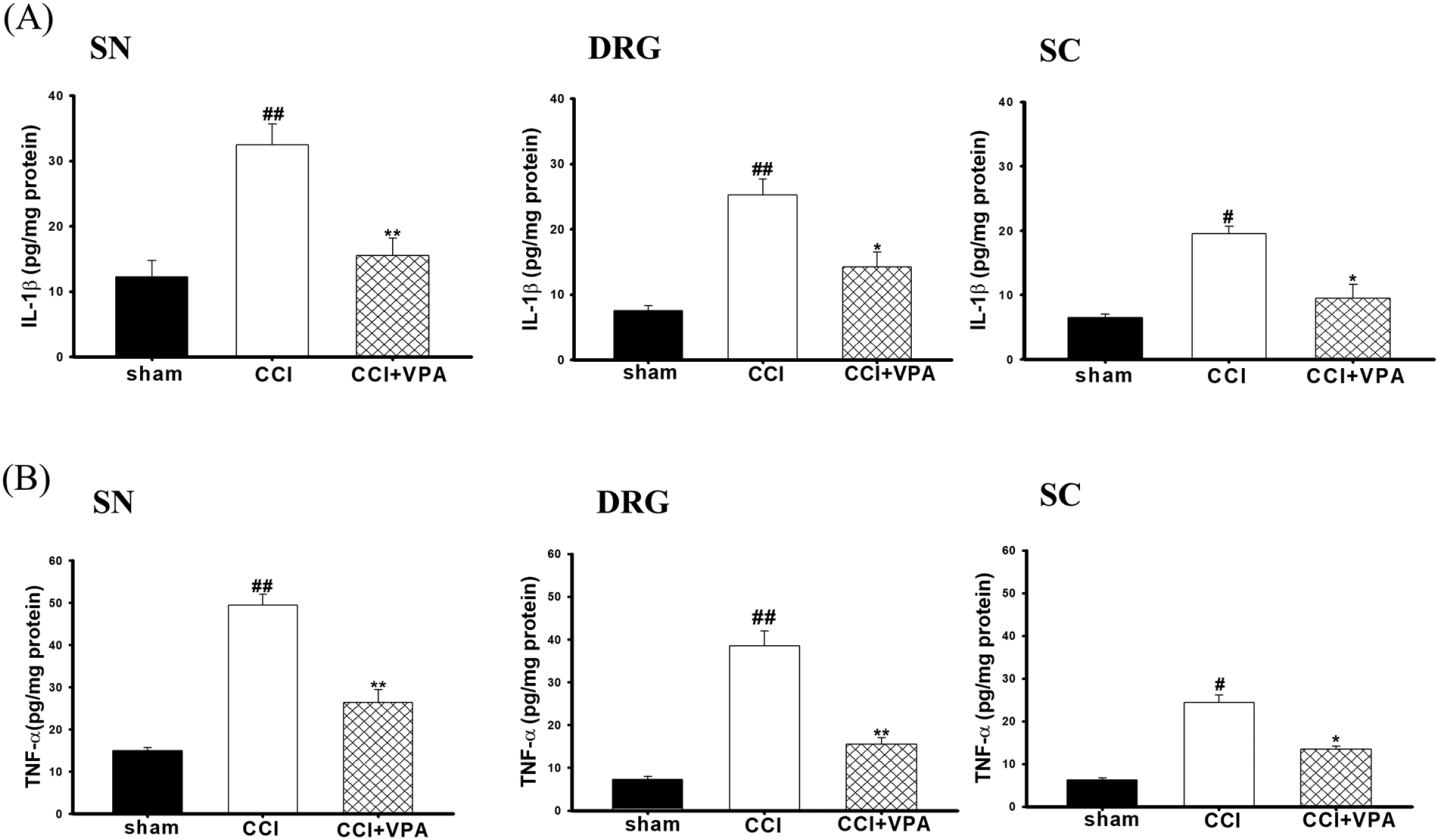
Effects of valproate (VPA, 300 mg/kg/day, i.p.) on elevated proinflammatory cytokine levels in sciatic nerve (SN), dorsal root ganglia (DRG) and spinal cord (SC) in CCI rats for 7 days. (**A**) IL-1β and (**B**) TNF-α levels in the SN, DRG and SC. Data represent the mean ± SEM for 6 rats per group. ^#^p < 0.05 and ^##^p < 0.01 compared with the sham group; ^*^p < 0.05 compared with the CCI group.

Since CCI-induced pain behaviors in the rat model were noticeable on POD 3 and most severe on POD 7, TNF-α and IL-1β were subsequently measured at the POD 7 time point. VPA effects on TNF-α and IL-1β in the SN, DRG and SC of CCI rats was examined by using commercial enzyme-linked immunosorbent assay (ELISA) kits. In the CCI group, IL-1β and TNF-α levels were significantly increased versus the sham group in all three tissues on POD 7. Notably, CCI-elevated cytokines were significantly lower in the CCI + VPA rats (Fig. 4).

### VPA decreased COX-2 and pGSK-3β immunofluorescence in the SN, DRG and SC

**Figure 5:**
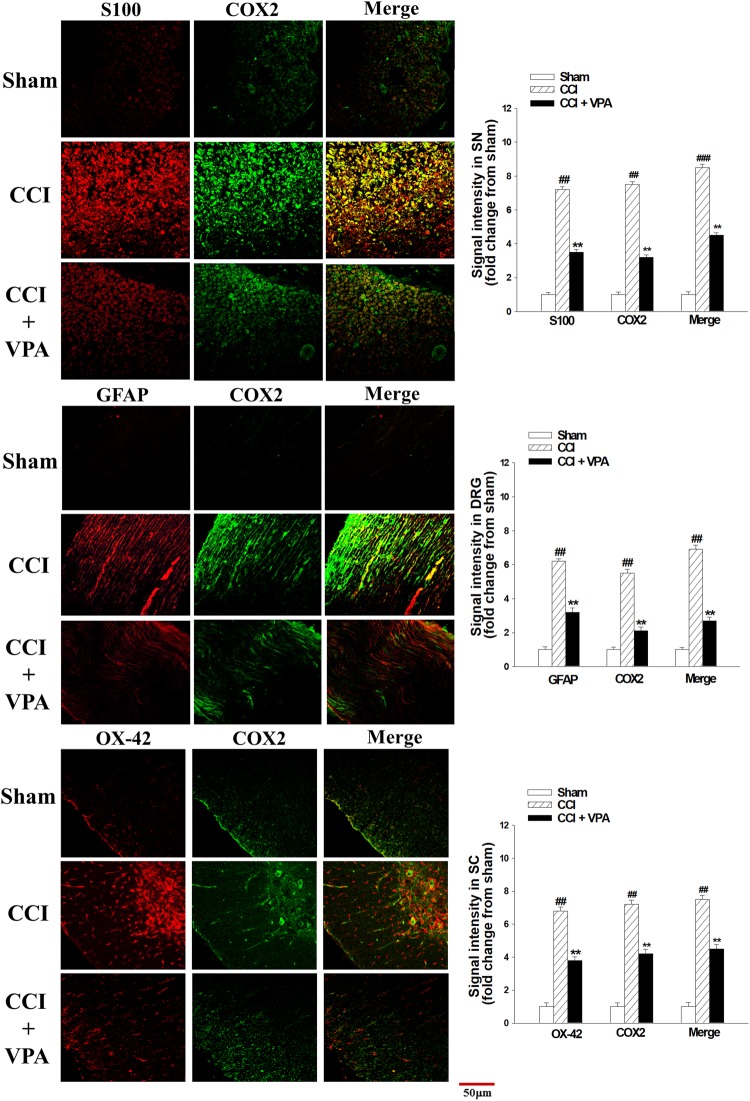
Double immunofluorescent staining for S100 (Schwann cell marker), GFAP (DRG satellite cell marker), OX-42 (spinal microglia marker) and COX-2 in the sciatic nerve (SN), dorsal root ganglia (DRG) and spinal cord (SC) on day 7 after chronic constriction injury (CCI). COX-2 protein expression was significantly increased in the CCI group compared with the sham and CCI + VPA groups. Administration of VPA attenuated CCI-enhanced COX-2 proteins. Quantitative fluorescence data are depicted. Data represent the mean ± SEM for 6 rats per group. ^##^p < 0.01 and ^###^*p* < 0.001 compared with the sham group; ^*^p < 0.05 and ^**^p < 0.01 compared with the CCI group. Scale bar, 50 μm; magnification, 400×.

**Figure 6:**
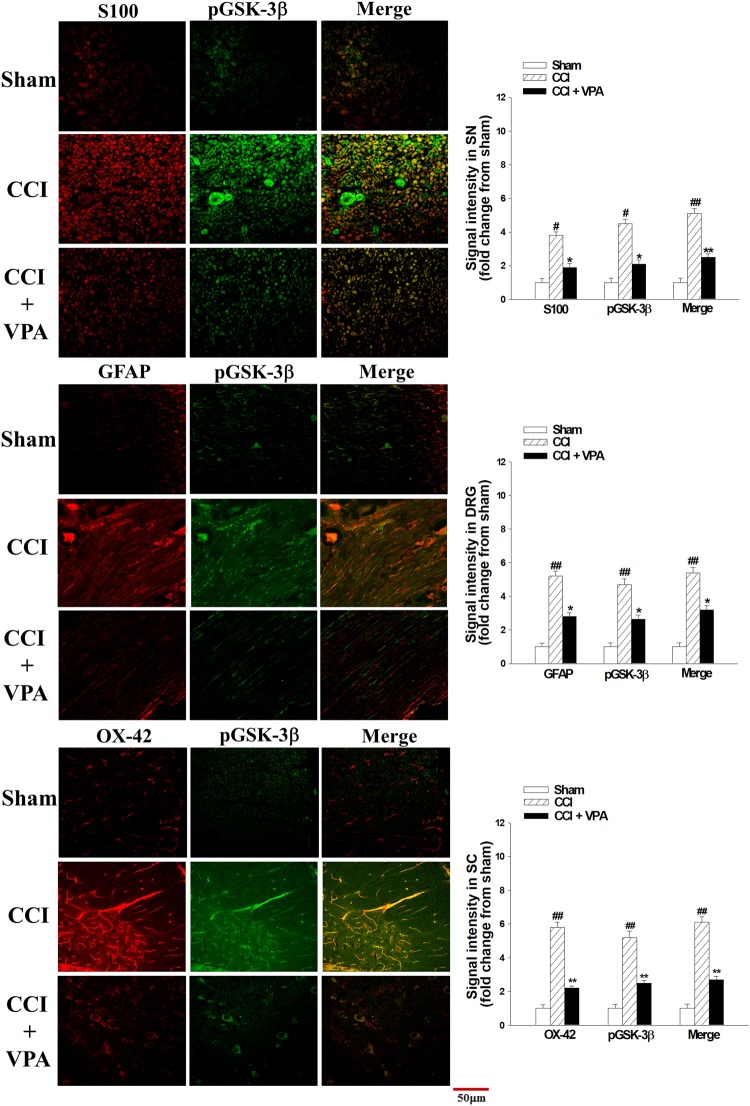
Double immunofluorescent staining for S100 (Schwann cell marker), GFAP (DRG satellite cell marker), OX-42 (spinal microglia marker) and pGSK-3β in the sciatic nerve (SN), dorsal root ganglia (DRG) and spinal cord (SC) on day 7 after chronic constriction injury (CCI). pGSK-3β protein expression was significantly increased in the CCI group compared to sham and CCI + VPA groups. Administration of VPA attenuated CCI-enhanced pGSK-3β proteins. Quantitative fluorescence data are depicted. Data represent the mean ± SEM for 6 rats per group. ^#^p < 0.05 and ^##^p < 0.01 compared with the sham group; ^*^p < 0.05 compared with the CCI group. Scale bar, 50 μm; magnification, 400×.

Double immunofluorescence staining further verified COX-2 and pGSK-3β involvement in peripheral nerve injury. CCI rats had markedly increased levels of COX-2 (Fig. 5) and pGSK-3β (Fig. 6) protein in Schwann cells (S100-positive), DRG (GFAP-positive) satellite cells and OX-42-positive spinal microglia on POD 7. In these glial cells, the CCI + VPA group demonstrated significantly decreased expression levels of COX-2 and pGSK-3β compared with the upregulated expression in the CCI group.

## Discussion

The major findings of this study were as follows: (1) VPA administration in the rat CCI model significantly improved neuropathic pain behavior after thermal and mechanical stimuli-induced hyperalgesia and allodynia. (2) The proinflammatory cytokines decreased after VPA administration in the SN, DRG and SC of rats in the CCI model. (3) VPA treatment downregulated the expression of pNFκB/iNOS/COX-2 and reduced the expression of pAkt/pGSK-3β in the SN, DRG and SC of rats following CCI.

In adult patients, therapeutic VPA concentrations between 50–100 μg/ml are commonly prescribed for epilepsy, and 50–125 μg/ml for mania^19^. A 400 mg/kg intraperitoneal injection in rodents produces a 150 μg/ml plasma concentration at 8 hr^20^. Many studies achieved HDAC or GSK-3 inhibition in brain or other tissues with 300 to 400 mg/kg^20–23^. We therefore used 300 mg/kg VPA, and this dose showed a preventive effect on CCI-induced neuroinflammation and neuronal death in rats. However, our experimental design had some limitations. We did not determine the effect of VPA on a sham group. Also, bilateral CCI surgery cannot separate the nerve tissues into ipsilateral and contralateral sides^6^. There is a previous study showing both neuroprotective effects and improved motor and cognitive functions from VPA in a traumatic brain injury rodent model^20^. Our study addressed the biochemical mechanisms of sensory function in a CCI pain model. A comprehensive explication of VPA’s full motor and cognitive effects remains to be accomplished.

After CCI surgery, the rats exhibited abnormal posture of the injured hind paw, in addition to frequent licking and trembling of the injured hind paw, implicating spontaneous pain. Those nociceptive behaviors, including hyperalgesia and allodynia, are similar to neuropathic symptoms and signs in chronic pain patients^24^. A hyperalgesic condition was obvious from POD 3 to POD 14 (the last observation), with the peak on POD 7. Our results suggested that once-daily administration of VPA significantly improved CCI-induced neuropathic pain behaviors.

Generally, uninjured peripheral nerves are composed of resident macrophages, fibroblasts, Schwann cells and the wrapping glia of the PNS. After the axons are damaged, Schwann cells outnumber resident macrophages roughly 10 to 1^25^. In the CCI model, the sciatic nerve is loosely ligated and chronically constricted by catgut. Transmission neurons are excited and subsequently the axons degenerate, which leads to demyelination attributable to sensitization of nociceptors and ectopic excitability of afferent neurons. This process exhibits the development and maintenance of peripheral neuropathy^26^. Schwann cells are the dominant glia of the PNS and they differentiate into. myelinating and non-myelinating cells^27,28^. Microglia are recognized as tissue-resident macrophages in the CNS, and satellite glial cells are important in the establishment of physiological pain in sensory ganglia, particularly in dorsal spinal ganglia^29^. Astrocytes and microglia are the main neural cells involved in neuron inflammation. There are several reports indicating that glial cells have a similar inflammatory role within the CNS^30,31^.

NFκB has a central role in the gradual expansion and maintenance of pain hypersensitivity induced by nerve injury^32^. In this study, pNFκB rose markedly after nerve injury from POD 3 to POD 14 and then decreased after VPA treatment. Behavioral findings, combined with reduced expression of pNFκB in the SN, DRG and SC after VPA administration suggest that VPA can be developed as a valuable therapeutic drug for the control of neuropathic pain resistant to ordinary analgesics. We also found that COX-2, the key enzyme inducing inflammatory pain, was reduced after VPA administration in our CCI model. Neuronal COX-2 has been identified as a NFκB target gene^33^. It was reported that chronic treatment of rats with VPA downregulated NFκB DNA-binding activity and subsequently reduced COX-2 mRNA expression in rat frontal cortex^33,34^. Additionally, nitric oxide (NO) is a unique molecule involved in many physiological processes in the CNS. The anti-hyperalgesia status was related to a significant comparative decrease of many proinflammatory and pronociceptive mediators, including NO levels^35^. This study showed that VPA appeared to alleviate iNOS in all three tissues. Since NO is an important signal molecule in neuropathic pain, its molecular mechanisms and interaction with VPA in the pain pathway in the CCI model merit further investigation.

pAKT/pGSK-3β is a crucial factor in many aspects of neural cell regulation, including neurogenesis, neural stem cell proliferation, neuronal differentiation, neural cell death, and gliogenesis^36^. pAKT/pGSK-3β was found to have a pro-apoptotic activity toward neuronal death in several models, including DNA damage and amyloid β protein-induced neurotoxicity. Overexpression of active pAKT/pGSK-3β has been confirmed to promote neuronal apoptosis^37^. The behavioral tests in our study showed that CCI rats had the shortest latency of thermal paw withdrawal on day 7, indicating that behavioral change-related neuropathic pain reached its maximum severity on day 7 following CCI, in accordance with the change in pAKT/pGSK-3β expression. Therefore, we hypothesized that pAKT/pGSK-3β may be important in the pathophysiology of neuropathic pain development and maintenance.

The release of proinflammatory cytokines (TNF-α and IL-1β) are strongly correlated with the pathogenesis of neuropathic pain. TNF-α produces either beneficial or deleterious characteristics through its proinflammatory and pro-apoptotic effects in different cell types^38^. TNF-α is also involved in the induction of apoptosis through binding to TNF receptor (TNFR) in Schwann cells^39^. IL-1β is an endogenous ligand of IL-1 receptor (IL-1R) and a powerful proinflammatory cytokine^40^. By activating IL-1R, IL-1β also contributes to hyperalgesia and the formation of peripheral and central sensitization^40^. IL-1β can mobilize neuronal calcium and stimulate microglia or astrocytes to generate proinflammatory mediators such as TNF-α through NFκB activation^40,41^. Activation of TNF-α/IL-1β-mediated NFκB activity releases several inflammatory mediators, which may contribute to high levels of pain^42,43^. In addition, TNF-α and IL-1β are intensified in chronic pain models and expressed in glial cells and primary sensory neurons^24^. Upregulation of TNF-α results in the intensification of neuronal death in the rat spinal cord, and some studies have demonstrated that TNF-α was overexpressed around the injury area after spinal cord injury^44^. We found that VPA significantly reduced pNFκB expression and release of proinflammatory cytokines in the SN, DRG and SC in this study. Our findings suggest that VPA could be used to inhibit inflammatory and neuropathic pain from injury to the peripheral nerves.

**Figure 7:**
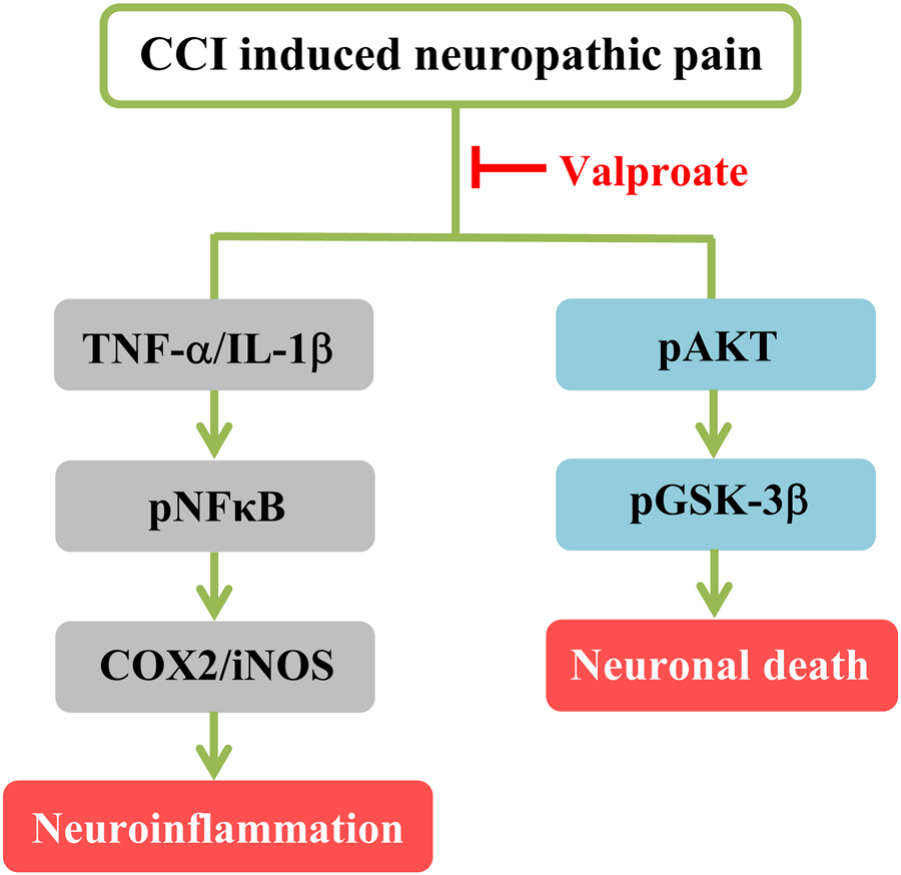
Scheme summarizing the proposed mechanism by which VPA decreases NFκB/COX2/iNOS-mediated neuroinflammation and pAKT/pGSK-3β-mediated neuroapoptosis in a rat model of CCI.

In this study, VPA reduced the level of inflammatory proteins (pNFκB, iNOS and COX-2), pro-apoptotic proteins (pAKT/AKT and pGSK-3β/GSK-3β), proinflammatory cytokines (TNF-α and IL-1β), and the immunoreactivity of COX-2 and pGSK-3β proteins (Fig. 7). These results indicate that VPA may protect against peripheral nerve injury-induced neurogenic inflammation and neuronal cell death. We suggest that the antiepileptic drug VPA might be useful for treating patients suffering from painful peripheral neuropathy.

## Materials and Methods

### Animals

Male Sprague-Dawley rats, weighing 250–300 g, were purchased from National Laboratory Animal Center (BioLASCO Taiwan Co., Ltd, Taipei, Taiwan), habituated in the animal facility for a week and housed in constant temperature, humidity and light cycle with ad libitum food and water. This study was approved by the Animal Care and Use Committee of Kaohsiung Medical University and followed the guidelines of the National Institute of Health for the use of the experimental animals.

### Chronic constriction injury (CCI) surgery

Bilateral CCI surgery was performed as described previously for the CCI animal model of neuropathic pain^6,45–47^. Briefly, sixty rats were anesthetized with 40 mg/kg, i.p. pentobarbital sodium, and the sciatic nerve exposed by blunt dissection of the biceps femoris muscle, and dissected and freed proximal to the sciatic trifurcation. Three 4-0 chromic catgut ligatures^45–48^ were loosely tied around the sciatic nerve until the rat’s hind limb briefly twitched. Both sides of the sciatic nerve were handled using the same procedure. Sham-operated rats had the same surgical procedures without ligature placement.

### Drug administration and pain-related behavioral tests

Rats were given an hour to adapt to the laboratory environment before behavioral testing. The paw withdrawal response to thermal stimulus was performed 1 day before CCI in rats. Then, rats were intraperitoneally administered vehicle or VPA (Sanofi-Aventis Co., Paris, France) once daily after CCI. We used 300 mg/kg/day of sodium VPA^22,23^. Neuropathic pain remains measurable 2 weeks after the procedure^49^. The behavioral tests consisted of mechanic allodynia and thermal hyperalgesia before surgery (day −1) and 1, 3, 7 and 14 days following surgery. The infrared radiant heat method was used to assess the thermal hyperalgesia. Rats were put in an elevated plexiglass cage for 1 hour to adapt to the environment. The glass surface was maintained at 30 °C. Thermal hyperalgesia was evaluated by placing a radiant heat source just beneath the right hind paw to measure the PWL using a plantar analgesiometer (IITC360, Woodland Hills, CA) with a 30 second cutoff time. The PWL was calculated as described previously^45–47,50^. Mechanical stimuli-induced pain sensitivity was measured by using a series of von Frey filaments (from 2.5 to 50 g). PWT was detected by dynamic plantar aesthesiometer (Ugo Basile, Varese, Italy). Each von Frey test was repeated 3 times at 5 min intervals, and the average force inducing reliable withdrawals was recorded as the threshold^45–47,51^.

### Western blot analysis and nuclear extraction

The specimens from the different time points were collected, frozen and stored at −80 °C until required. Samples were homogenized with ice-cold lysis buffer (Roche protease inhibitor cocktail tablet/10 ml Thermo Scientific T-PER Tissue Protein Extraction Reagent) and then centrifuged at 20,000 g at 4 °C for 30 min. Nuclear and cytoplasmic protein extractions (Nuclei Isolation Kit, Sigma-Aldrich, Saint Louis, MO) were prepared from each side of the proximal SN, DRG (L4-L6) and SC (L4-L6) from sham, CCI and CCI + VPA groups. Next, separate nuclear and cytoplasmic fractions were used for Western blot analysis. The nuclear fractions were used to detect pNFκB exclusively. The procedures and analyses of Western blot were performed as previously described^45–47^. The primary antibodies of GSK-3β, pGSK-3β (1:500 dilution, Cell Signaling Technology, Danvers MA), AKT (1:1000 dilution, Cell Signaling Technology), pAKT (Ser473, 1:500 dilution, Cell Signaling Technology), pNFκB (1:500 dilution, Cell Signaling Technology), COX-2 (1:500 dilution, Abcam London, UK), iNOS (1:1000 dilution, Santa Cruz Biotechnology, Santa Cruz CA) and β-actin (1:2000 dilution, Sigma-Aldrich, Saint Louis MO) were used in this experiment.

### ELISA analysis of TNF-α and IL-1β

TNF-α and IL-1β concentrations were quantified by Quantikine ELISA kits (R&D Systems, Minneapolis MN) according to the manufacturer’s instructions^45–47^. The proximal SN, DRG (L4-L6) and SC (L4-L6) were collected at POD 7, homogenized with protease inhibitors in lysis buffer, and the insoluble pellet separated from the supernatant by centrifugation. The total protein concentration in the supernatant was estimated by the Bradford method, with the concentration of TNF-α or IL-1β measured by color intensity. Sample values were read after establishing a standard curve.

### COX-2 and iNOS immunofluorescence assay

The proximal SN, DRG (L4-L6) and SC (L4-L6) were removed at POD 7 following CCI in rats. Double immunofluorescent staining was conducted as we previously described^45,46^. Specimens were embedded in optimal cutting temperature (OCT) compound and frozen to decrease the variation in experimental procedures. Sections 10 microns thick were cut and incubated with the primary antibodies anti-S100 Schwann cell marker (Millipore, Temecula CA), anti-GFAP DRG satellite cell marker (Millipore), anti-OX-42 spinal microglia marker (Millipore), anti-pGSK-3β antibodies at 1:500 dilution (Cell Signaling Technology, Danvers MA) and anti-COX-2 at 1:200 dilution (Abcam, London UK), followed by species-specific fluorescent secondary antibodies (Jackson ImmunoResearch Laboratories Inc., West Grove PA) at 22 °C for 1 hour. The staining images acquired by confocal microscope (Olympus Fluoview FV1000, Olympus Optical Co., Tokyo, Japan).

## Data analysis and statistics

Data are expressed as the mean ± SEM (n = 6 per group). Results were analyzed by one-way analysis of variance (ANOVA) with Tukey-Kramer pairwise comparison when appropriate for *post hoc* analysis. *p* < 0.05 was considered statistically significant.
